# Lhermitte Sign as a Presenting Symptom of Thoracic Spinal Pathology: A Case Study

**DOI:** 10.1155/2015/707362

**Published:** 2015-08-02

**Authors:** Adam Hills, Mazen Al-Hakim

**Affiliations:** ^1^Class of 2015, OUWB School of Medicine, Rochester, MI 48309, USA; ^2^Department of Neurology, William Beaumont Health System, Troy, MI 48085, USA

## Abstract

A 54-year-old male with ankylosing spondylitis presented with complaints of progressively worsening bilateral leg weakness and difficulty ambulating of 2-week duration. He also felt a sharp, electric, shock-like sensation radiating from his lower back into his legs upon flexing the trunk. There was no history of trauma or other inciting events within the 2 weeks prior to presentation. Thoracic MRI at this visit showed a three-column fracture at T11-T12. He underwent spinal fusion surgery and within 2 days after surgery the radiating electrical sensation with spinal flexion had completely resolved.

## 1. Introduction

Lhermitte sign was originally described in 1924 as a symptom consisting of an electric shock-like sensation radiating down the spine when the neck is flexed [1]. This sensation often travels down the trunk and into the lower limbs and can be associated with various other sensory components such as hyper- or hypoesthesia.

Thoracic lesions result in a Lhermitte sign much less often than cervical lesions, and with that in mind we present this case as a clinical reminder of thoracic pathology as a potential source for this symptom. Here we describe a previously healthy 54-year-old man with ankylosing spondylitis who developed Lhermitte sign as a presenting symptom shortly after sustaining an acute injury to his thoracic spine.

## 2. Case Presentation

A 54-year-old male with a history of progressive ankylosing spondylitis presented to our hospital with complaints of worsening bilateral leg weakness and difficulty ambulating. He also described a shooting, electric sensation that began in his lower back and radiated laterally to his hips and down his legs whenever he flexed his trunk, such as when transferring from a seated to standing position. A skiing accident occurred approximately 2 months prior to presentation during which the patient sustained a fracture dislocation of his right shoulder. Imaging at that time included X-rays of the right shoulder, in addition to imaging of the thoracic and lumbar spine. These images were unremarkable other than degenerative changes consistent with the patient's previous diagnosis of ankylosing spondylitis. Several weeks after the accident he began to experience an intermittent, sharp pain in his lower back that radiated anteriorly around his abdomen at the T10 level. He had been to both his primary care and physical medicine and rehabilitation physicians after the accident, and they had followed him up for further medical management and physical therapy.

Approximately 2 weeks prior to admission, the patient began to experience the intermittent shock-like sensation and worsening bilateral lower extremity weakness. He described his weakness as not only a perceived loss of muscular strength but also an excessive fatigue resulting in the inability to complete physical activities that had previously been routine for him. Two days before presenting to the hospital, the patient was unable to lift himself off an exercise mat after the completion of a physical therapy appointment and required the assistance of his wife and son to help him return to his vehicle. He subsequently developed a wide-based and ataxic gait with frequent loss of balance and near falls. Pertinent negatives throughout the course of this patient's presentation include any loss of bowel or bladder function, loss of consciousness, dizziness, light-headedness, vertigo, headache, numbness, tingling, burning, or other neurological symptoms other than those already described.

The patient's past medical history included ankylosing spondylitis initially diagnosed at the age of 25 and hypertension treated with amlodipine. He had undergone an umbilical hernia repair in 2004. He was a previous smoker, having quit in 2007, and denied alcohol or drug abuse. Upon presenting to the EC, the patient received a preliminary laboratory workup consisting of a CMP, CK, ESR, and CBC, all of which were unremarkable other than elevated WBC of 10.2 and ESR of 31 consistent with his underlying inflammatory condition. A CT of the brain and cervical spine revealed no signs of acute infarct, hemorrhage, or fracture. Due to the nature of the patient's symptoms an MRI was ordered of the same regions and also interpreted with unremarkable results. The patient was subsequently admitted for further neurologic and orthopedic workup.

Upon examination, the patient demonstrated hip flexor and foot dorsiflexion weakness bilaterally with vague sensory disturbances of the left and right anterior thigh, as described by the patient. An ataxic gait was also present, and the patient was unable to complete tandem gait testing due to loss of balance. Position and vibration sense, heel to shin testing, and reflexes were preserved throughout the lower extremities. Interestingly, flexion of the neck did not elicit any symptoms; however, the sudden shooting pain located in the lower back, hip, and legs presented when the patient was asked to stand from a lying position. The patient was otherwise intact neurologically. There was no focal vertebral tenderness to palpation over the cervical or thoracic spine.

**Figure 1:**
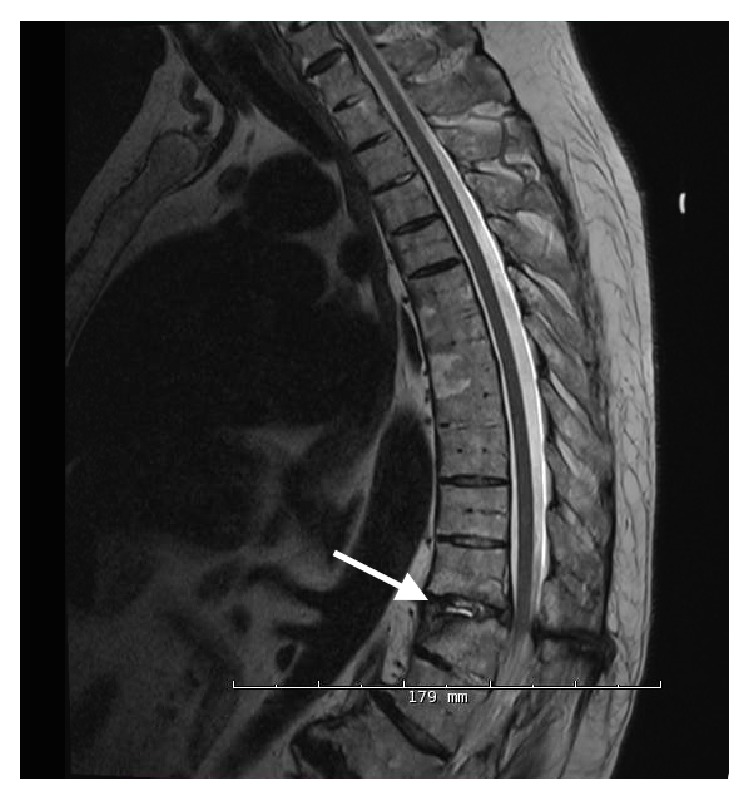
T2 thoracic MRI showing focal signal abnormality at the T11-T12 level (white arrow) with associated edema and loss of disc height, in addition to moderate spinal canal stenosis.

Given the patient's clinical examination and symptoms we were most concerned with an underlying progressive pathology of the spinal cord, whether it be of neurologic, orthopedic, or multifactorial origin. We had also not ruled out metabolic, inflammatory, or infectious etiologies at that point without more extensive laboratory testing. Our differential diagnosis at the time included cervical or thoracic spondylotic myelopathy, vertebral disc herniation, thoracic vertebral fracture, spinal stenosis, transverse myelitis, chronic inflammatory demyelinating polyneuropathy, multiple sclerosis, thoracic or lumbosacral radiculopathy, pernicious anemia, malignancy, or an infectious condition such as tabes dorsalis. Our primary concern was to first investigate a potential acute spinal cord injury, so we ordered STAT cervical and thoracic MRIs to evaluate any underlying physical abnormalities that may help explain the patient's overall presentation. The T2 thoracic MRI revealed a three-column vertebral fracture at the T11-T12 level that was associated with significant edema surrounding the spinal cord and loss of vertebral body height, which had resulted in moderate spinal canal stenosis at that level (Figure 1). Later that day the patient underwent an uncomplicated T10-L1 laminectomy and fusion with instrumentation to stabilize the vertebral column. Within 2 days post-op the radiating electrical sensation with spinal flexion had disappeared, and the patient was able to stand and walk without loss of coordination or balance. He was discharged several days later able to stand and walk without assistance and felt that he had regained much of his previous lower-body strength by that point. He continued to be compliant with his physical therapy regimen and reported his symptoms completely resolved by the time of his 1-month follow-up visit with the surgeon.

## 3. Discussion

Lhermitte sign was first described as a subjective electric sensation that radiated down the spinal column and into the extremities of patients with cervical demyelinating lesions. Initially it had been most commonly associated with multiple sclerosis; however, any intrinsic or extrinsic cervical cord lesion is capable of producing this sign. Cervical spondylosis, disc herniation, trauma, spinal cord malignancies, vitamin B12 deficiency, cisplatin toxicity, and radiation myelotoxicities have all been previously described as etiologies of Lhermitte sign [2–4]. Although this sign has typically been associated with cervical pathology, there are isolated reports of thoracic lesions resulting in symptoms consistent with Lhermitte sign in the literature (Table 1), [5–9].

Nearly all of these reports regarding thoracic lesions and Lhermitte sign are related to spinal cord compression due to malignancy, rather than an injurious event. In addition, several cases have been reported which describe the onset of Lhermitte sign after significant head and/or neck trauma; however, to our knowledge there is no published report of Lhermitte sign in a patient with a history of distal thoracic trauma [10].

In our patient, the lack of any abnormal sensations upon cervical flexion as well as the pattern of symptoms after the injury led us to believe that any spinal pathology would likely be located in the thoracolumbar region. The fact that truncal flexion could elicit the shock-like sensation, as well as the combination of ataxic gait, bilateral lower extremity weakness, and unremarkable imaging of the head and neck, further strengthened our convictions. Historically, thoracic etiologies of Lhermitte sign have been much less common than cervical ones and as a result have the potential to be overlooked in the course of a clinical evaluation. Given the relative infrequency with which this particular symptom manifests, we present this case as a brief clinical example of Lhermitte sign as a rare, but possible, presentation of spinal cord pathology within the thoracic region.

## Figures and Tables

**Table 1:** Reported cases of Lhermitte sign due to isolated thoracic lesions.

Case report authors	Description of symptoms	Inciting event
Baldwin and Chadwick [5]	Electric shock-like sensation radiating down the back and into the legs, associated with tingling and numbness in the legs and occasional urinary incontinence.	Cavernous hemangioma leading to extradural compression at T5.

Rogers [6]	Sensory disturbance radiating to the anterior thighs upon cervical neck flexion.	Metastatic prostate carcinoma leading to spinal cord compression at T5.

Liveson and Zimmer [7]	Electric shock-like sensation radiating down the spine and occasionally into the toes upon trunk flexion.	Gibbus deformities of unknown cause at T6 and T7 with anterior extradural compression of the spinal cord.

Broager [8]	Sensation of intense paresthesia in both anterior thighs upon cervical flexion, accompanied by weakness and decreased sensation in the legs.	Metastatic prostate carcinoma leading to spontaneous T5 vertebral fracture.

Ventafridda et al. [9]	Lhermitte sign present in the spine and posterior lower limbs upon cervical flexion, accompanied by weakness and paresthesias in the lower limbs.	Metastatic breast sarcoma at T3-T4 causing extradural spinal cord compression.

Ventafridda et al. [9]	Lhermitte sign radiating from the spine to the anterior thighs with cervical flexion, accompanied by weakness in the lower limbs, abdominal paresthesias, and impaired sphincter control.	Metastatic breast tumor at T7-T8 with epidural metastatic deposits.

Ventafridda et al. [9]	Lhermitte sign present in the spine and posterior lower limbs upon cervical flexion, accompanied by weakness in the lower limbs and hypesthesia at the thoracic level.	Metastatic lung tumor at T2 causing extradural spinal cord compression.
